# Sex-stratified early biomarker model identifies lactate as the key predictor of in-hospital deterioration in acute heart failure

**DOI:** 10.3389/fcvm.2026.1717901

**Published:** 2026-01-20

**Authors:** Mohammadreza Akbarian Khorasgani, Pouriya Katouzi, Melika Khalifeh Hadi, Linsong Leng, Aliasgar Taha Burhanpurwala, Xiangjuan Liu

**Affiliations:** 1 Department of Cardiology, Qilu Hospital of Shandong University, Jinan, China; 2 State Key Laboratory for Innovation and Transformation of Luobing Theory, Qilu Hospital of Shandong University, Jinan, China; 3 Key Laboratory of Cardiovascular Remodeling and Function Research, Ministry of Education, National Health Commission and Chinese Academy of Medical Sciences, Qilu Hospital of Shandong University, Jinan, China; 4 Department of Internal Medicine, Dr. Sulaiman Al Habib Hospital, Dubai, United Arab Emirates

**Keywords:** acute heart failure, biomarkers, deterioration, lactate, neutrophil-to-lymphocyte ratio, NT-ProBNP, sex differences, troponin I

## Abstract

**Introduction:**

Early identification of patients at risk for deterioration during hospitalization for acute heart failure (AHF) is essential for guiding intensive monitoring and advanced therapies. Biomarkers such as NT-proBNP and troponin I are routinely used, yet their comparative prognostic performance—particularly when stratified by sex—remains uncertain. Other emerging biomarkers, including lactate and the neutrophil-to-lymphocyte ratio (NLR), have also been linked to adverse outcomes, but their value relative to established cardiac markers has not been clearly defined.

**Methods:**

We conducted a retrospective cohort study using de-identified electronic medical records from 2010 to March 2025 at a tertiary care center. Patients aged ≥16 years with clinician-documented AHF and available admission biomarkers were eligible. The primary endpoint was a composite of in-hospital death, mechanical ventilation, extracorporeal membrane oxygenation (ECMO), or intra-aortic balloon pump (IABP). Broad and strict endpoints were examined in sensitivity analyses. Multivariable logistic regression models, sex-stratified analyses, and penalized regressions with bootstrap resampling were performed.

**Results:**

Among 143 eligible patients (81 men, 62 women), the primary endpoint occurred in 46.9%. In our cohort, women experienced a slightly higher crude rate of in-hospital deterioration compared with men (48.4% vs. 45.7%). Lactate was the most robust predictor across all models, with an odds ratio of 9.38 (95% CI 2.47–35.63; *p* = 0.001) per log10 increase and a clear dose–response (event rates 39.8%, 55.2%, and 85.7% across lactate strata ≤2, 2–4, and >4 mmol/L; *p*-trend = 0.002). In sex-stratified models, NT-proBNP (OR 2.87; *p* = 0.029) and lactate (OR 28.98; *p* = 0.003) were significant in men, while no biomarker reached significance in women. NLR predicted outcomes in the non-HFrEF subgroup. Model performance was modest (AUC ∼0.71–0.73) but calibration was good. Findings remained consistent in winsorized and bootstrap sensitivity analyses.

**Conclusions:**

In this single-center AHF cohort, lactate emerged as the most consistent early biomarker associated with in-hospital deterioration, with stronger prognostic performance than the other evaluated cardiac markers. Sex-stratified and phenotype-specific findings (NT-proBNP and lactate in men, NLR in non-HFrEF) were exploratory and did not show significant sex–biomarker interaction. These results support incorporating lactate into early risk stratification and highlight the need for larger multicenter validation studies.

## 1 Introduction

Acute heart failure (AHF) represents one of the most common and life-threatening emergencies encountered in cardiovascular medicine, accounting for substantial morbidity, mortality, and healthcare utilization worldwide (1). Despite advances in pharmacological and device-based therapies, in-hospital outcomes for patients admitted with AHF remain poor, with deterioration events such as death, mechanical ventilation, and need for advanced circulatory support occurring in a significant proportion of cases (2). Early identification of patients at high risk for in-hospital deterioration is therefore critical for optimizing triage, guiding intensive monitoring, and informing timely therapeutic interventions.

Biomarkers have become integral to the diagnosis and management of heart failure. Natriuretic peptides such as NT-proBNP are established diagnostic and prognostic markers (3–5), while troponins are routinely applied to detect myocardial injury (6, 7). Inflammatory indices, including the neutrophil-to-lymphocyte ratio (NLR), have gained attention as inexpensive predictors of adverse outcomes, particularly in non-ischemic and preserved EF phenotypes (8–10). Lactate, reflecting systemic hypoperfusion and metabolic stress, is widely used in critical care, but its prognostic utility in the broader population of AHF patients remains less well defined (11, 12). Although each of these biomarkers captures distinct aspects of the pathophysiology of AHF, their comparative and combined predictive performance for short-term in-hospital deterioration is not fully established.

Another dimension often overlooked in biomarker research is the influence of sex. Biological differences between men and women, including myocardial structure, hormonal milieu, and renal clearance, may alter biomarker expression and prognostic thresholds. Prior studies have suggested that natriuretic peptides and inflammatory markers may behave differently across sexes, but evidence in the specific setting of AHF remains sparse (13). Addressing this gap is important for developing sex-informed approaches to precision medicine in heart failure.

In this context, we conducted a retrospective cohort study of hospitalized patients with AHF to evaluate the prognostic performance of four routinely measured biomarkers—NT-proBNP, troponin I, NLR, and lactate—for predicting in-hospital deterioration. We specifically incorporated sex-stratified analyses and subgroup evaluations by heart failure phenotype and acute myocardial infarction status. Our aim was to determine which early biomarkers provide the strongest prognostic value and whether sex-specific differences exist, thereby informing future strategies for individualized risk stratification in AHF.

## 2 Materials and methods

### 2.1 Study design and setting

This retrospective cohort study was conducted using de-identified electronic medical records (EMRs) from Qilu Hospital, a high-volume tertiary center affiliated with Shandong University. The study period extended from 2010 to March 31, 2025. Retrospective EMR-based designs were chosen because they enable efficient access to a large number of well-characterized patients, minimize recall bias, and reflect real-world clinical practice. The protocol for this study was approved by the Ethics Committee of Qilu Hospital of Shandong University, and the study was conducted in accordance with the principles of the Declaration of Helsinki. As this was a retrospective cohort study and the follow-up was performed by phone, the ethics committee permitted verbal consent. No direct patient contact occurred, and no identifiable information was accessed.

### 2.2 Study population and eligibility criteria

Patients were eligible for inclusion if they were aged 16 years or older and had an index hospitalization with clinician-documented acute heart failure (AHF), identified by either a coded or written diagnosis at admission or discharge. Only those with early biomarker data available within 24 h of admission and complete outcome ascertainment during the index hospitalization were considered. AHF was defined based on clinician-documented diagnosis at admission and discharge, supported by EMR notes and coding. No uniform biomarker cutoff (e.g., BNP) or echocardiographic threshold was enforced, which may have allowed diagnostic heterogeneity. In routine practice at our center, clinicians diagnose AHF based on acute onset or worsening of typical heart failure symptoms and signs (e.g., dyspnea, orthopnea, pulmonary congestion, peripheral edema) requiring hospitalization, supported by chest imaging, echocardiography, and natriuretic peptide testing when available. This real-world approach was preserved in our study and the resulting diagnostic heterogeneity is explicitly acknowledged as a limitation. Patients also needed to have all core covariates available for risk adjustment to be included. The primary endpoint was defined as a composite of in-hospital death, mechanical ventilation, extracorporeal membrane oxygenation (ECMO), or intra-aortic balloon pump (IABP). Detailed and consistent information on vasopressor and inotrope use (initiation, dose, and duration) was not systematically available across the 15-year EMR period; therefore, vasopressor therapy could not be reliably operationalized and was not incorporated into the primary endpoint. Transfers to the intensive or coronary care unit (ICU/CCU) were incorporated only in sensitivity analyses.

Exclusion criteria were defined to minimize misclassification and ensure diagnostic specificity. Patients were excluded if they had a non-HF primary diagnosis clearly documented, such as pure COPD/asthma or primary pneumonia/ARDS without heart failure attribution. Cases where decompensation was attributed to primary non-cardiac septic shock were also excluded. Additional exclusions included incomplete medical records, missing key biomarker data, duplicate admissions, or transferred-in cases without initial laboratory information.

A total of 250 patients were screened under these criteria, of whom 143 met the eligibility standards and were included in the final analysis.

### 2.3 Variables and definitions

Forty-one variables were extracted for each patient, covering demographic, clinical, hemodynamic, laboratory, and outcome domains:

- **Demographics & comorbidities**: age, sex, BMI, height, weight, smoking, alcohol use, hypertension, diabetes mellitus, CAD/MI history, chronic kidney disease, COPD, atrial fibrillation.
- **Clinical signs & vitals**: heart rate, systolic/diastolic blood pressure, respiratory rate.
- **Blood gas parameters**: pH, PaO₂, PaCO₂, HCO₃^−^.
- **Cardiac function**: LVEF, HF type (HFrEF <40%, HFmrEF 40%–49%, HFpEF ≥50%), Killip class (AMI patients only).
- **Biomarkers**: NT-proBNP, troponin I (TnI), neutrophil count, lymphocyte count, neutrophil-to-lymphocyte ratio (NLR), lactate, ALT, total bilirubin, creatinine, sodium, potassium.
- **Outcomes**: length of stay, ICU/CCU transfer, in-hospital death, mechanical ventilation (MV), ECMO, IABP.

All biomarker and arterial blood gas measurements were taken from the earliest available results within 24 h of admission.

**Endpoints:**

- **Primary endpoint**: composite of in-hospital death, MV, ECMO, or IABP.
- **Broad endpoint**: primary + ICU/CCU transfer.
- **Strict endpoint**: death, ECMO, or IABP only.

### 2.4 Data collection and processing

All 250 screened patients were initially assessed for completeness. Patients with missing core variables (e.g., admission biomarkers or outcome data) were excluded during the eligibility process, leaving 143 patients with complete records. For the final analytic cohort, no variables used in the main analyses had missing values (0% missingness across the 41 collected variables). As recommended by STROBE, missingness patterns were reviewed, and complete-case analysis was therefore applied without imputation.

Data were manually extracted from EMRs and entered into standardized Excel spreadsheets with dropdown menus to minimize transcription errors. Two independent reviewers cross-checked all entries to ensure accuracy. Extreme laboratory values were flagged, and each was manually verified against the original EMR. Confirmed extreme values were retained as true clinical findings. Derived indices such as BMI and NLR were calculated using standardized formulae. This rigorous workflow was adopted to ensure diagnostic specificity, reduce misclassification, and improve reproducibility.

### 2.5 Statistical analysis

Descriptive statistics, classical (unpenalized) logistic regression, Hosmer–Lemeshow tests, and Brier scores were performed in IBM SPSS Statistics v29 (IBM Corp.). Penalized logistic regression and all bootstrap procedures were performed in R v4.3.2 (R Foundation) using glmnet (penalized models), pROC (ROC/AUC with confidence intervals), and boot (bootstrap resampling). Continuous variables are summarized as median [IQR] or mean ± SD, and categorical variables as counts (percent). To address right-skew, biomarkers were log10-transformed (lg_*).

- **Single-biomarker performance** was evaluated with ROC analysis against the primary endpoint, and Youden-optimal cut-points were reported.
- **Multivariable models included**: Model A (biomarkers only) and Model B (biomarkers + age, HF phenotype, creatinine). Model performance was summarized with AUCs (bootstrap 95% CIs), Hosmer–Lemeshow goodness-of-fit, Brier scores, and calibration plots based on deciles of predicted risk.
- **Prespecified subgroup analyses** included sex, HF phenotype (HFrEF <40% vs. non-HFrEF [HFmrEF 40%–49% + HFpEF ≥ 50%]), and AMI with Killip class (I–IV; I as reference).
- **Sensitivity analyses** comprised alternative endpoints (broad, strict), winsorization of outliers (99th percentile), penalized regression, and bootstrap internal validation. All tests were two-sided with *α* = 0.05.

To mitigate small-sample/events-per-variable (EPV) limitations and reduce overfitting, we fitted L2-penalized (ridge) logistic regression with the same predictors as the unpenalized models. The regularization parameter *λ* was selected by 10-fold cross-validation minimizing binomial deviance (glmnet default). Because ridge does not perform variable selection, adjusted odds ratios (ORs) were obtained by exponentiating the penalized coefficients; corresponding 95% CIs and *p*-values were derived from 2,000 stratified bootstrap resamples (see below). Conventional (unpenalized) estimates for Model A and Model B are reported alongside penalized estimates as a robustness check.

For internal validation, we used stratified bootstrap resampling (B = 2,000, stratified by outcome) to obtain 95% CIs for (i) AUCs of single biomarkers and multivariable models, (ii) penalized ORs, and (iii) calibration summaries where applicable. For AUCs, CIs were computed from the bootstrap percentile distribution. For penalized ORs, exponentiated coefficients from each bootstrap replicate were used to form percentile CIs and two-sided bootstrap *p*-values. Hosmer–Lemeshow tests were calculated for the unpenalized models, and calibration was also assessed visually.

Transformation formulas were: lg_ntprobnp = log10(NT-proBNP + 0.001), lg_tni = log10(troponin I + 0.001), lg_nlr = log10(NLR + 0.001), and lg_lact = log10(lactate + 0.001). Pre-specified lactate strata were ≤2, 2–4, and >4 mmol/L. EPV was calculated for each model; penalization and bootstrap were planned *a priori* for models with EPV <10 (14). Because several models—particularly sex-stratified and HF-phenotype subgroup models—had <10 events per predictor, we treated penalized estimates as the primary results, considered subgroup findings as exploratory and hypothesis-generating, and reported unpenalized ORs only for transparency.

## 3 Results

### 3.1 Proliferation of biomarkers

#### 3.1.1 Distribution of biomarkers

All four biomarkers (NT-proBNP, troponin I, NLR, and lactate) were log10-transformed to address right-skewed distributions. The transformed variables showed improved symmetry, although troponin I and lactate retained extreme outliers (Supplementary Figure S1). This justified subsequent winsorization and outlier sensitivity analyses. NT-proBNP and NLR displayed near-normal distributions, whereas lactate clustered around 1 mmol/L with a long right tail, consistent with heterogeneous metabolic stress among patients.

#### 3.1.2 Baseline characteristics

A total of 143 patients (81 males, 62 females) with acute heart failure were analyzed (Table 1). The median age was 66 years (IQR 54–73), with no significant difference by sex. Biomarker distributions showed sex-specific patterns: NT-proBNP concentrations were higher in women (7,133 ng/L [IQR 1,513–20,519]) compared to men [4,444 ng/L (IQR 1,728–14,6000)], while troponin I levels were substantially higher in men [124,640 ng/L (IQR 27,710–1,771,450)] than in women [42,075 ng/L (IQR 615–178,653)]. NLR and lactate medians were similar between sexes. Among liver and renal markers, ALT was higher in men, whereas bilirubin and creatinine showed no clear sex differences. As raw values were highly skewed, log10-transformed variables (lg_ntprobnp, lg_tni, lg_nlr, lg_lact) were used in subsequent regression models.

**Table 1:** Baseline characteristics of the study cohort overall and stratified by sex.

Variable	Overall	Male	Female
AGE	66.00 (54.00–73.00)	65.00 (53.00–72.00)	67.00 (55.00–75.00)
NT-PROBNP (NG/L)	4,960.00 (1,616.00–16,086.50)	4,444.00 (1,728.00–14,600.00)	7,132.50 (1,512.50–20,519.00)
TROPONIN (NG/L)	56,450.00 (12,450–635,390)	124,640.00 (27,710–1,771,450)	42,075.00 (615.00–178,652.50)
NLR	5.09 (2.90–9.56)	5.23 (3.31–8.63)	4.19 (2.59–15.03)
LACTATE (MMOL/L)	1.50 (1.00–2.30)	1.50 (0.95–2.30)	1.40 (1.00–2.27)
ALT (U/L)	21.00 (12.00–43.50)	25.00 (13.00–54.00)	17.50 (12.00–28.00)
TOTAL BILIRUBIN (µMOL/L)	13.00 (8.20–21.20)	12.90 (8.70–26.70)	13.20 (7.78–18.77)
CREATININE (µMOL/L)	93.00 (69.50–150.00)	92.00 (76.00–159.00)	93.50 (67.00–142.50)
NA + (MMOL/L)	135.00 (132.00–139.00)	135.19 ± 5.69	134.79 ± 5.88
K + (MMOL/L)	3.80 (3.50–4.30)	3.80 (3.50–4.20)	3.95 ± 0.71
LOG10(NT-PROBNP)	3.70 (3.21–4.21)	3.65 ± 0.59	3.85 (3.18–4.31)
LOG10(TROPONIN)	4.74 (4.07–5.76)	5.10 (4.44–6.25)	4.59 (2.69–5.24)
LOG10(NLR)	0.74 ± 0.41	0.71 ± 0.36	0.62 (0.41–1.18)
LOG10(LACTATE)	0.15 (−0.02–0.36)	0.18 (−0.05–0.36)	0.15 (0.00–0.36)

Baseline characteristics of the study cohort overall and stratified by sex. Continuous variables are presented as median (interquartile range) or mean ± SD as indicated. Log10-transformed variables were used for regression analyses.

#### 3.1.3 Event rates

Among the 143 included patients, in-hospital deterioration occurred in 67 cases (46.9%) according to the primary composite definition of death, mechanical ventilation, ECMO, or IABP (Table 2). Of the 67 primary endpoint events, 40 were mechanical ventilation, 15 in-hospital deaths, 7 intra-aortic balloon pump (IABP) uses, and 5 extracorporeal membrane oxygenation (ECMO) initiations (some patients experienced more than one). Thus, mechanical ventilation accounted for the majority of deterioration events. As detailed in the exclusion criteria (Section 2.2), patients with pure COPD/asthma, primary pneumonia/ARDS without HF attribution, or primary non-cardiac septic shock were excluded, so all MV events occurred in the context of clinician-diagnosed AHF. Nevertheless, concomitant pulmonary infection or obstructive lung disease may have contributed to respiratory failure in some cases. Using the broader definition, which additionally included ICU transfer, the event rate was 67.8% (97/143). When applying the strict definition (death, ECMO, or IABP only), 40 patients (28.0%) experienced deterioration. These three outcome definitions were used for primary and sensitivity analyses.

**Table 2:** Event counts and rates for three definitions of in-hospital deterioration.

Definition	n_events	n_total	event_rate_%
det_primary (primary deterioration)	67	143	46.85
det_broad (broad deterioration)	97	143	67.83
det_strict (strict deterioration)	40	143	27.97

Event counts and rates for three definitions of in-hospital deterioration in patients with acute heart failure.

We next evaluated the discriminative performance of individual biomarkers for predicting in-hospital deterioration.

#### 3.1.4 Single-biomarker ROC analysis

Discriminative performance of individual biomarkers is summarized in Table 3. Lactate demonstrated the highest AUC (0.689), with an optimal cutoff of 1.50 mmol/L (sensitivity 68.7%, specificity 67.1%). NT-proBNP (cutoff 3,132 ng/L, AUC 0.615) and NLR (cutoff 4.32, AUC 0.613) showed modest discrimination, while troponin I provided limited predictive value (cutoff 55,140 ng/L, AUC 0.560). These results suggest that among single biomarkers, lactate carries the strongest early prognostic signal for in-hospital deterioration.

**Table 3:** Youden-optimal cutoffs and ROC performance metrics of single biomarkers.

Biomarker	log10_cutoff	raw_cutoff	AUC	Sensitivity	Specificity
lg_ntprobnp	3.495822	3,132.0	0.615	0.701	0.513
lg_tni	4.741467	55,140.0	0.560	0.582	0.566
lg_nlr	0.635844	4.3226	0.613	0.716	0.513
lg_lact	0.176381	1.50	0.689	0.687	0.671

Youden-optimal cutoffs and ROC performance metrics of single biomarkers for predicting in-hospital deterioration in acute heart failure.

To assess whether combining biomarkers improves prediction beyond single markers, we constructed multivariable logistic regression models.

#### 3.1.5 ROC analysis of biomarkers and combined model

**Figure 1:**
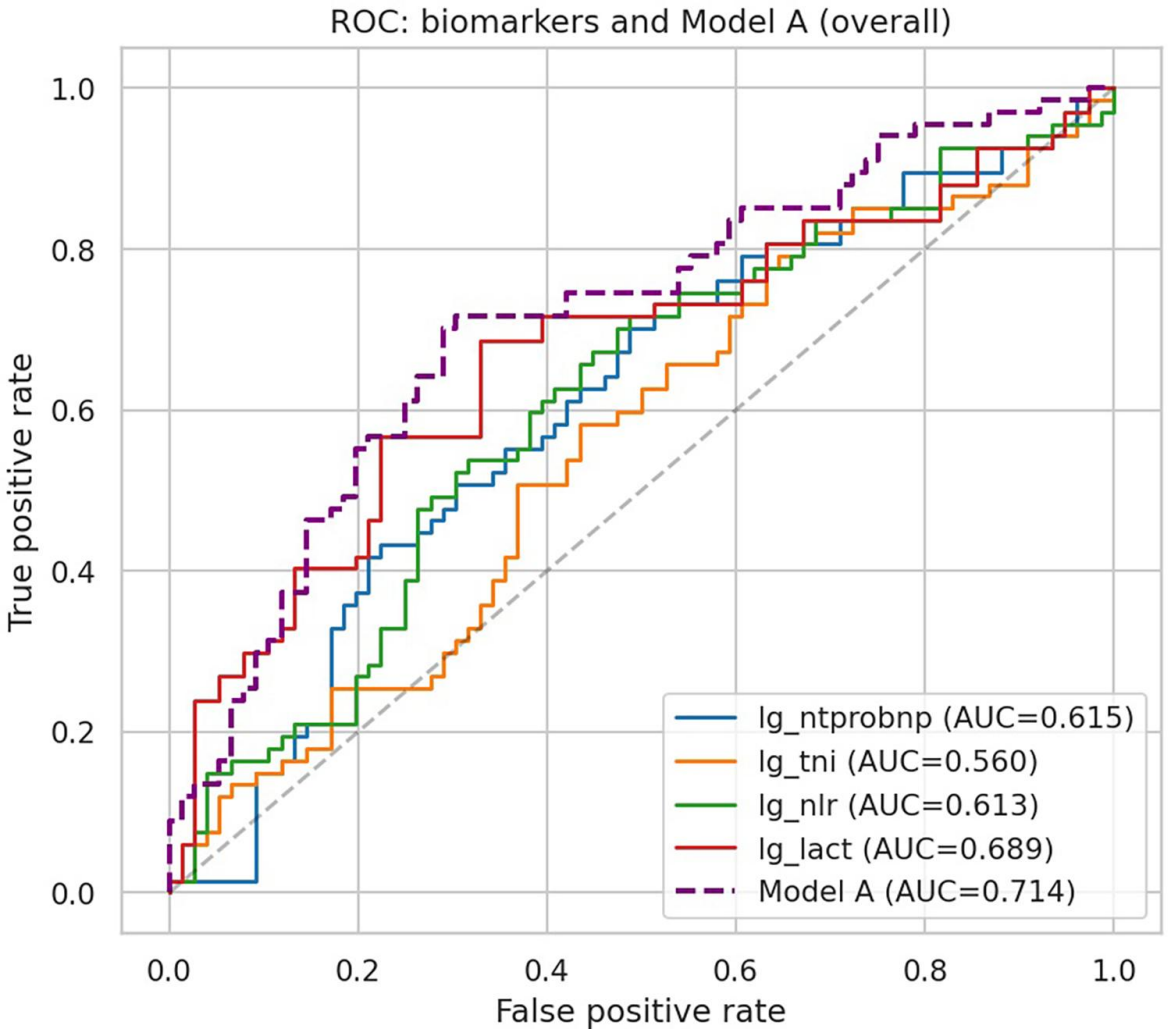
ROC curves of individual biomarkers and Model A. ROC curves of individual biomarkers and the combined biomarker model (Model A) for predicting in-hospital deterioration in acute heart failure.

The ROC curves for individual biomarkers and the overall multivariable biomarker model (Model A) are displayed in Figure 1. Among single biomarkers, lactate achieved the highest discrimination (AUC 0.689), followed by NT-proBNP (0.615) and NLR (0.613), while troponin I demonstrated limited value (AUC 0.560). The combined model integrating all four biomarkers yielded superior performance with an AUC of 0.714, exceeding any individual biomarker.

### 3.2 Multivariable logistic regression (Model A & Model B)

#### 3.2.1 Multivariable biomarker model (Model A)

**Figure 2:**
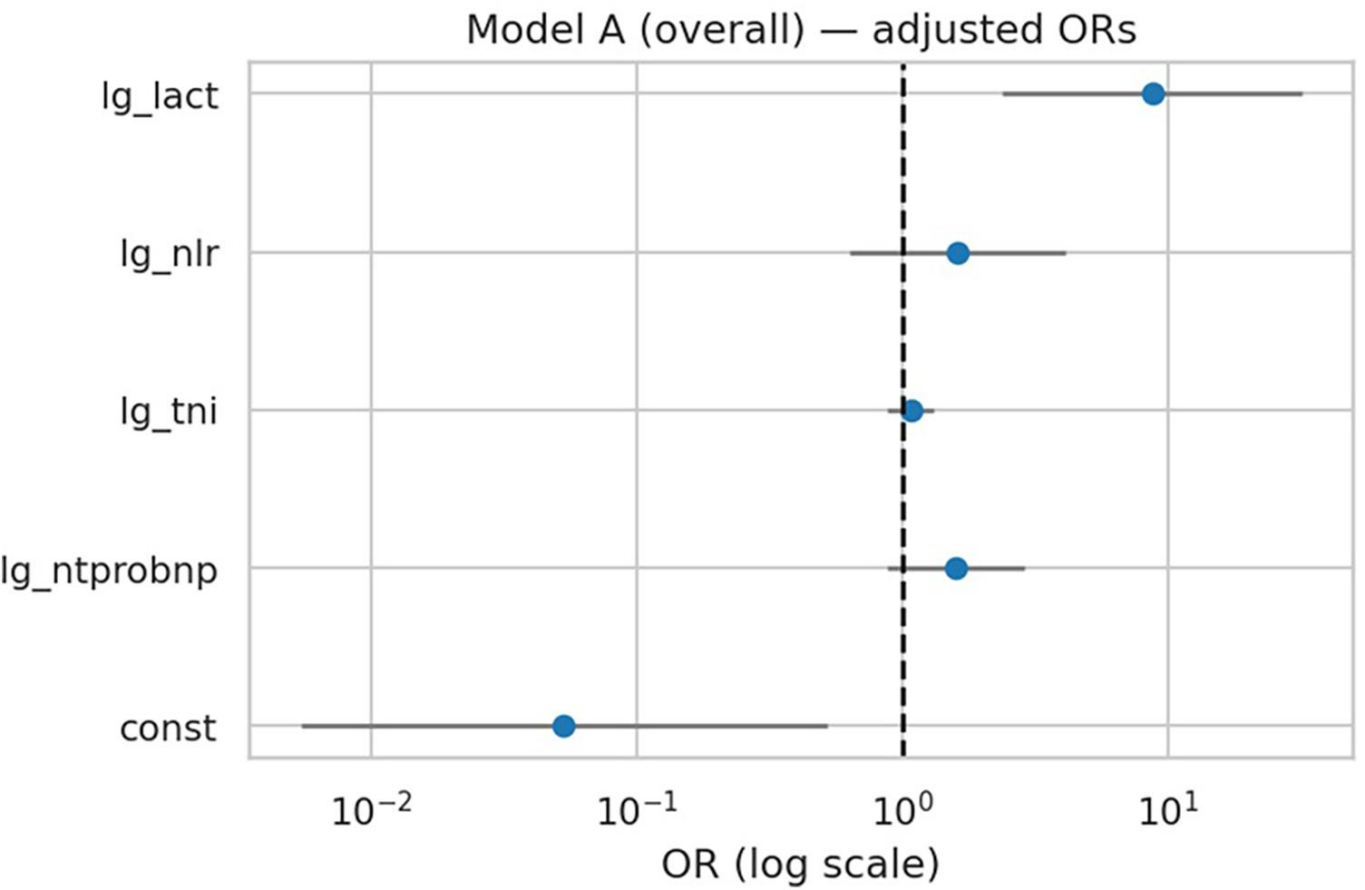
Forest plot of multivariable regression (Model A). Adjusted odds ratios (ORs) with 95% confidence intervals for biomarkers in Model A (overall). Lactate was the only independent predictor of in-hospital deterioration.

In the overall cohort, only lactate remained independently associated with in-hospital deterioration after mutual adjustment of biomarkers (OR 8.74, 95% CI 2.37–32.16; *p* = 0.001). NT-proBNP (OR 1.59, *p* = 0.13), troponin I (OR 1.07, *p* = 0.48), and NLR (OR 1.62, *p* = 0.31) were not significant predictors (Table 4, Figure 2). The multivariable model achieved an AUC of 0.714 (95% CI 0.629–0.784) with acceptable calibration (Hosmer–Lemeshow *p* = 0.20).

**Table 4:** Multivariable logistic regression (Model A: biomarkers only).

Term	Coef	OR	95% CI (low)	95% CI (high)	*p*
const	−2.932498	0.053	0.0054	0.524	0.01197
lg_ntprobnp	0.460968	1.586	0.875	2.875	0.1289
lg_tni	0.070897	1.073	0.883	1.305	0.4770
lg_nlr	0.484239	1.623	0.637	4.133	0.30997
lg_lact	2.167667	8.738	2.374	32.162	0.001113

Model diagnostics: AUC 0.714 (95% CI 0.629–0.784 by bootstrap); Hosmer–Lemeshow *p* = 0.204 (good fit).

#### 3.2.2 Adjusted biomarker model (Model B)

**Figure 3:**
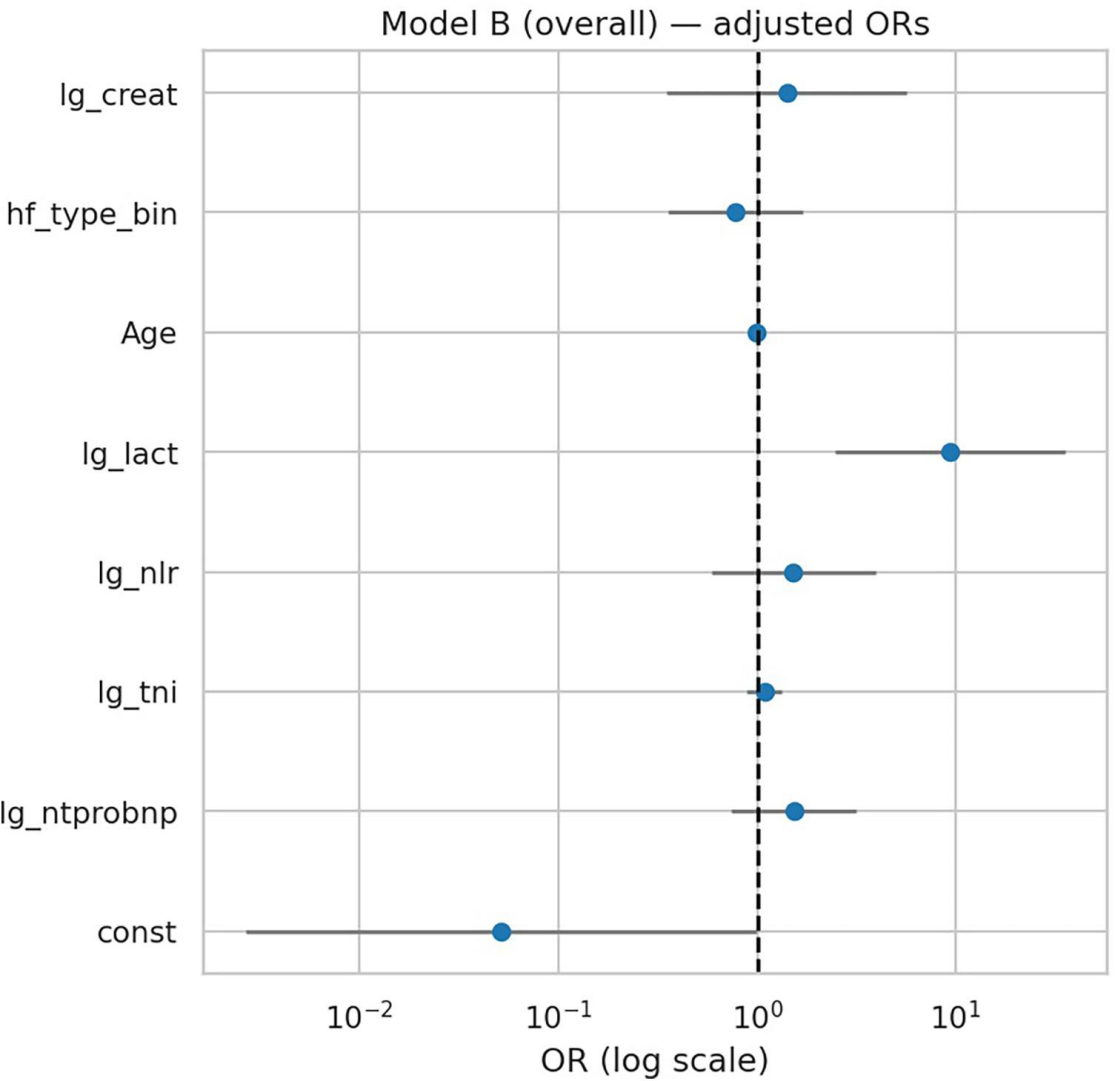
Forest plot of multivariable regression (Model B). Adjusted odds ratios (ORs) with 95% CIs for biomarkers and clinical covariates in Model B (overall). Only lactate remained independently associated with deterioration.

After additional adjustment for age, HF phenotype, and creatinine, lactate remained the only significant predictor of in-hospital deterioration (OR 9.38, 95% CI 2.47–35.63; *p* = 0.001) (Table 5, Figure 3). However, penalized regression suggested a more conservative effect (OR 4.70, bootstrap 95% CI …), indicating that the unpenalized estimate may overstate the true magnitude. NT-proBNP, troponin I, NLR, age, HF phenotype, and creatinine were not independently associated with outcome. Model performance was modestly improved compared with Model A (AUC 0.725 vs. 0.714), with good calibration (Hosmer–Lemeshow *p* = 0.63).

**Table 5:** Multivariable logistic regression (Model B: biomarkers + clinical covariates).

Term	Coef	OR	95% CI (low)	95% CI (high)	*p*
const	−2.975830	0.051	0.0026	0.9836	0.04873
lg_ntprobnp	0.423661	1.528	0.742	3.147	0.2506
lg_tni	0.082637	1.086	0.890	1.326	0.4174
lg_nlr	0.415584	1.515	0.585	3.927	0.3925
lg_lact	2.239059	9.384	2.472	35.627	0.001004
age	−0.006989	0.993	0.971	1.016	0.5417
hf_type_bin	−0.247003	0.781	0.358	1.706	0.5355
lg_creat	0.340313	1.405	0.349	5.663	0.6322

AUC 0.725 (95% CI 0.636–0.798), slightly better than Model A; HL *p* = 0.627 → good calibration.

#### 3.2.3 Model performance and calibration

**Figure 4:**
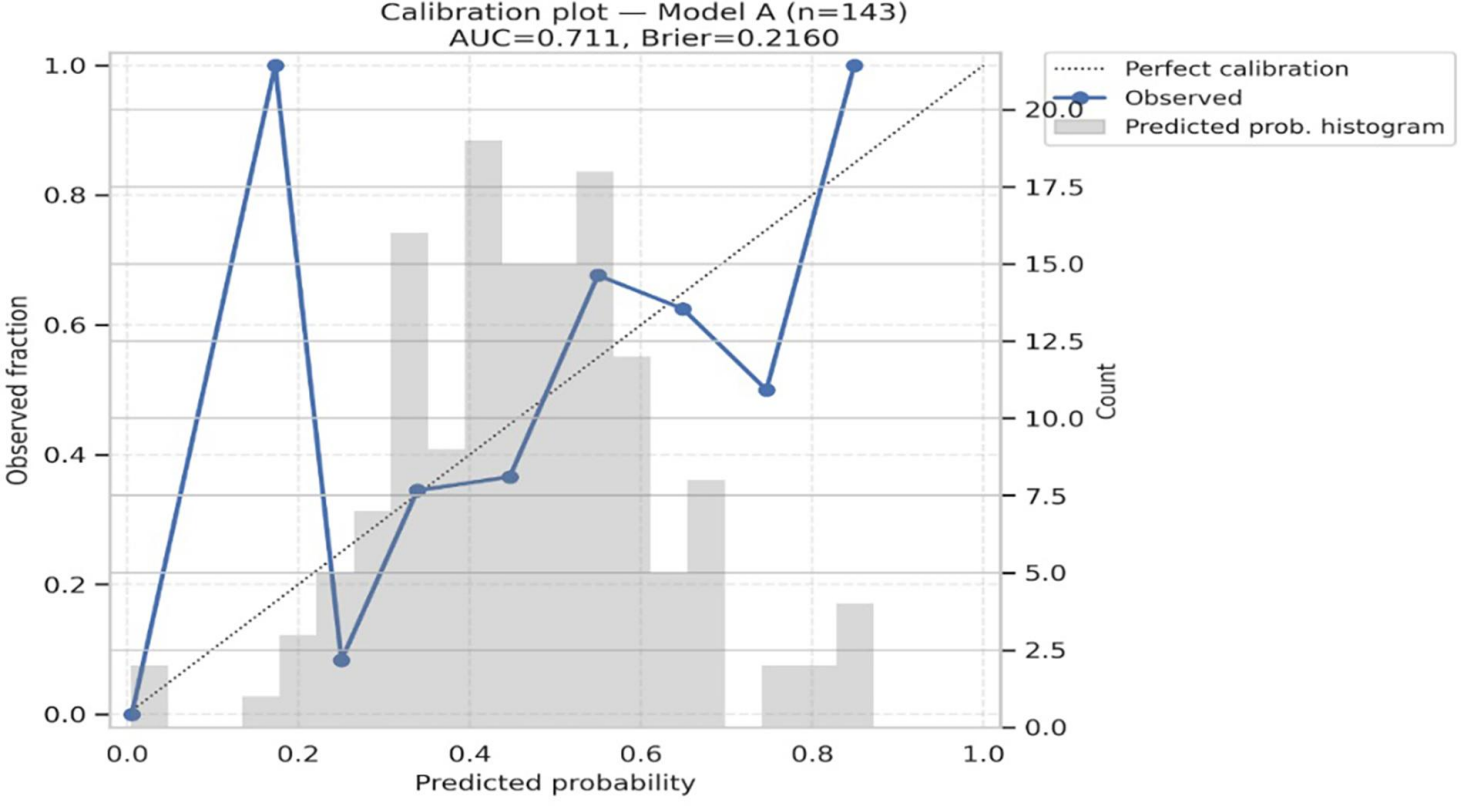
Calibration plot for Model A. AUC = 0.711; brier score = 0.2160. Hosmer–Lemeshow χ² = 4.384, *p* = 0.821. The diagonal line indicates perfect calibration; circles show observed fraction vs. predicted probability in 10 bins.

**Figure 5:**
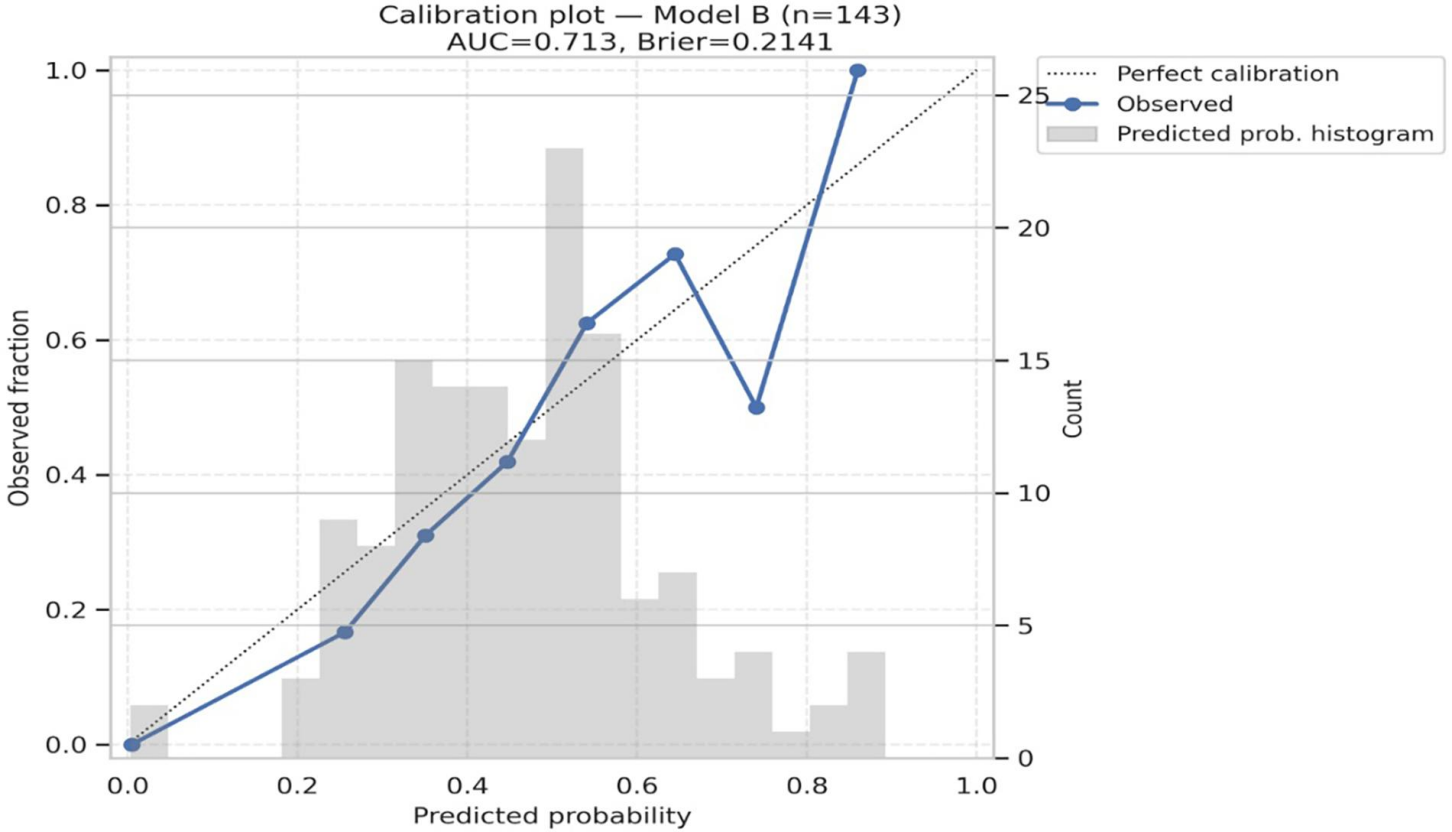
Calibration plot for Model B. AUC = 0.713; brier score = 0.2141. Hosmer–Lemeshow χ² = 8.084, *p* = 0.425. The diagonal line indicates perfect calibration; circles show observed fraction vs. predicted probability in 10 bins.

Model A (biomarkers only) achieved an AUC of 0.711 (95% CI 0.640–0.806), while Model B (biomarkers plus age, HF phenotype, and creatinine) achieved an AUC of 0.713 (95% CI 0.672–0.816). Both models demonstrated reasonable probability accuracy with Brier scores of 0.216 and 0.214, respectively. Calibration was good for both models, with non-significant Hosmer–Lemeshow tests (*p* = 0.821 for Model A, *p* = 0.425 for Model B), and calibration plots showed close alignment between predicted and observed event rates (Table 6, Figures 4, 5). Although model calibration was good by Hosmer–Lemeshow tests (*p* > 0.2) and Brier scores (∼0.21), these metrics should be interpreted cautiously, as the Hosmer–Lemeshow test has limited reliability in small samples, and a Brier score close to 0.25 indicates performance only modestly better than chance. These findings indicate that although overall discrimination was modest, the models produced well-calibrated risk estimates.

**Table 6:** Models' discrimination & calibration.

Model	n_complete_case	n_events	AUC_point	AUC_boot_CI	Brier_score	Hosmer–Lemeshow *χ*²	Hosmer–Lemeshow *p*
Model A	143	67	0.711	(0.640, 0.806)	0.2160	4.384	0.821
Model B	143	67	0.713	(0.672, 0.816)	0.2141	8.084	0.425

Discrimination and calibration of biomarker models. Calibration plots for Model A (biomarkers only) and Model B (biomarkers + age, HF type, creatinine) show good agreement between observed and predicted deterioration risk. Both models had modest discrimination (AUC ∼0.71–0.72) and acceptable probability accuracy (Brier scores 0.21).

Given potential biological differences, we next analyzed biomarker performance stratified by sex.

### 3.3 Sex-stratified analyses

#### 3.3.1 Sex-stratified Model A (Model A coefficients by sex)

When analyses were stratified by sex (Supplementary Tables S1–S2), biomarker associations differed between men and women. In men (*n* = 81), both NT-proBNP (OR 2.87, 95% CI 1.11–7.42; *p* = 0.029) and lactate (OR 28.98, 95% CI 3.53–238.1; *p* = 0.003) were significant predictors of in-hospital deterioration. In women (*n* = 62), lactate showed a directional but non-significant association (OR 4.11, 95% CI 0.80–21.3; *p* = 0.091), and other biomarkers were not predictive. Model discrimination was higher in men (AUC 0.788, 95% CI 0.681–0.884) compared with women (AUC 0.647, 95% CI 0.523–0.765), despite non-significant formal interaction tests (Supplementary Tables S1–S3, Supplementary Figure S2). These findings suggest potential sex-specific differences in the prognostic performance of biomarkers.

#### 3.3.2 Sex interactions

Formal interaction testing between sex and biomarkers did not reveal statistically significant interactions (all *p* > 0.05; Supplementary Table S4). While NT-proBNP and lactate showed directional trends toward stronger associations in males, these did not meet conventional significance thresholds. Accordingly, sex-stratified analyses were reported for interpretability, but no definitive effect modification by sex can be concluded from the present data.

We further examined whether biomarker associations varied by heart failure phenotype (HFrEF vs. non-HFrEF).

### 3.4 Penalized regression and subgroup analyses

Penalized logistic regression with bootstrap resampling confirmed lactate as the most robust predictor of in-hospital deterioration across the overall cohort and within subgroups (Supplementary Table S5, Supplementary Figure S3). In the overall model, lactate was strongly predictive (OR 4.70, 95% CI 2.23–11.16; *p* < 0.001), whereas NT-proBNP, troponin I, and NLR were not significant. HF phenotypes were defined as: HFrEF (heart failure with reduced ejection fraction, LVEF <40%), HFmrEF (mid-range ejection fraction, 40%–49%), and HFpEF (preserved ejection fraction, ≥50%). For subgroup analysis, HFmrEF and HFpEF were combined as non-HFrEF. Within the HFrEF subgroup (*n* = 54), lactate remained the only significant biomarker (OR 4.24, *p* < 0.001) (Supplementary Table S6). In the non-HFrEF subgroup (*n* = 89), both lactate (OR 2.80, *p* = 0.004) and NLR (OR 2.39, *p* = 0.034) were significant predictors (Supplementary Table S7). These findings emphasize lactate as the most consistent and independent biomarker, with NLR showing context-specific predictive value in non-HFrEF patients.

Because acute myocardial infarction is a common cause of acute heart failure, we performed an additional analysis restricted to AMI patients with Killip classification.

### 3.5 AMI subset with Killip class

**Figure 6:**
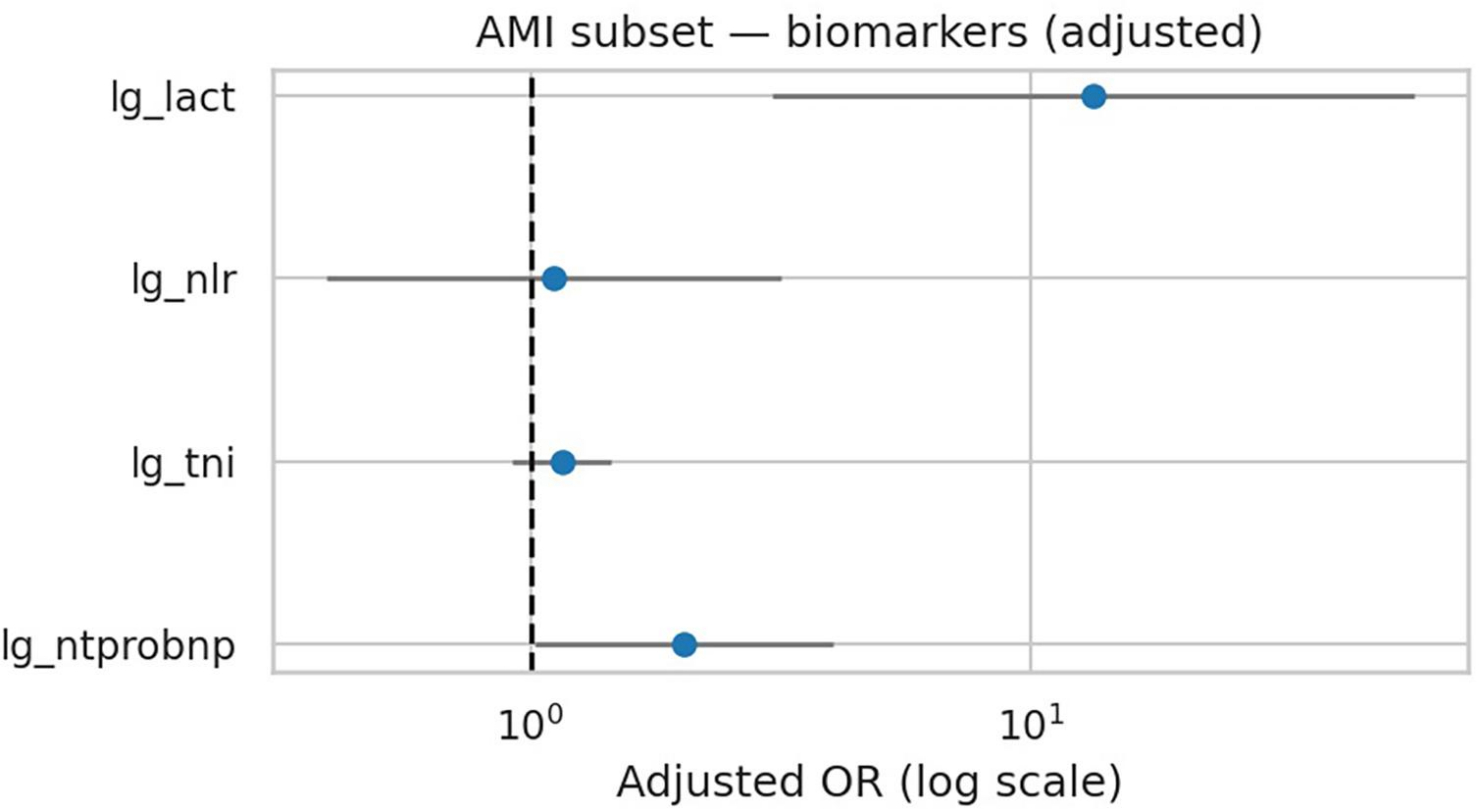
AMI subset — adjusted ORs for biomarkers (Model A + Killip). Lactate shows largest adjusted effect.

Killip class was recorded for AMI patients at admission, with Killip I (no heart failure) used as the reference group. Killip II–IV indicate progressively severe clinical heart failure. In the subgroup of 121 patients with AMI and documented Killip class, lactate remained the strongest independent predictor of deterioration (OR 14.44, 95% CI 3.26–63.99; *p* < 0.001), whereas NT-proBNP, troponin I, and NLR were not significant (Table 7, Figure 6). Killip class showed directional associations with higher deterioration risk but did not reach statistical significance in this dataset. Discrimination was good for both categorical and ordinal Killip models (AUC 0.761 and 0.752, respectively), and calibration plots confirmed reliable performance (Supplementary Figures S4–S5 in Supplementary Material). These results underscore the prognostic value of lactate even in AMI-associated acute heart failure.

**Table 7:** Multivariable regression in AMI subset with Killip class.

Term	Coef	OR	95% CI low	95% CI high	*p*
const	−4.545585	0.0106	0.000462	0.2439	0.00448
lg_ntprobnp	0.638420	1.8935	0.9334	3.8412	0.0769
lg_tni	0.149762	1.1616	0.9288	1.4526	0.1893
lg_nlr	0.043055	1.0440	0.3629	3.0035	0.9364
lg_lact	2.669954	14.4393	3.2585	63.9852	0.000439
Killip_2	0.805102	2.2369	0.3556	14.0714	0.3909
Killip_3	0.472002	1.6032	0.2668	9.6340	0.6059
Killip_4	1.092054	2.9804	0.5069	17.5254	0.22698

In the AMI subgroup, lactate remained the strongest independent predictor, even after accounting for Killip class. The combined model (Killip + lactate) gives one of the best AUCs seen in your study (∼0.76).

To explore the clinical utility of lactate, we analyzed deterioration rates stratified by lactate levels.

### 3.6 Lactate stratification and outcomes

Stratified analysis demonstrated a clear dose–response relationship between lactate level and in-hospital deterioration (Table 8). Patients with lactate ≤2 mmol/L had an event rate of 39.8%, compared with 55.2% in those with 2–4 mmol/L and 85.7% in those with >4 mmol/L. A significant trend was observed across strata (Spearman rho = 0.257, *p* = 0.002), highlighting the clinical relevance of lactate thresholds in early risk assessment.

**Table 8:** In-hospital deterioration rates stratified by admission lactate levels.

Lactate strata (mmol/L)	n_total	events	event_rate_pct
≤2	98	39	39.80
2–4	29	16	55.17
>4	14	12	85.71

In-hospital deterioration rates stratified by admission lactate levels. A significant dose–response trend was observed, with markedly higher event rates in patients with lactate >4 mmol/L.

### 3.7 Model stability (EPV analysis)

Events-per-variable (EPV) calculations are shown in Supplementary Table S8. The primary model (Model A) had adequate EPV (13.4), supporting stable regression estimates. In contrast, Model B (EPV 8.4) and subgroup models, particularly HFrEF (EPV 4.8), were below conventional thresholds, raising concerns about overfitting. To mitigate this, penalized logistic regression and bootstrap methods were applied for sensitivity analyses. The AMI subset was not analyzable due to insufficient events.

Finally, to ensure robustness, we conducted multiple sensitivity analyses including outlier handling, bootstrap validation, and missingness checks.

### 3.8 supplementary analyses

#### 3.8.1 Missingness for predictors in Models A and B

Missingness for all predictors and outcomes was negligible (0%), enabling complete-case analysis without imputation. Accordingly, the final analytic sample included all 143 patients (Supplementary Table S9).

#### 3.8.2 Supplementary sensitivity analysis

Winsorization of biomarkers at the 99th percentile (Supplementary Table S10) confirmed the robustness of the primary findings. Lactate remained the only significant biomarker associated with in-hospital deterioration (OR 8.80, *p* = 0.001), while NT-proBNP, troponin I, and NLR were not significant. These results demonstrate that the prognostic value of lactate was not driven by extreme outliers.

#### 3.8.3 Supplementary outlier robustness analysis

Comparison of original and winsorized Model A (Supplementary Table S11) demonstrated virtually identical results. Lactate remained strongly associated with deterioration (OR ≈ 8.8, *p* = 0.001), while NT-proBNP, troponin I, and NLR were non-significant. These findings confirm that the prognostic value of lactate was not dependent on extreme values.

#### 3.8.4 Supplementary bootstrap sensitivity analysis

In the winsorized Model A with 2,000 bootstrap resamples (Supplementary Table S12), lactate remained strongly associated with deterioration (OR 8.80, 95% CI 2.96–57.40; *p* < 0.001). NT-proBNP, troponin I, and NLR were not significant. These results further confirm the robustness of lactate as the primary biomarker predictor.

## 4 Discussion

### 4.1 Principal findings

In this retrospective cohort study of 143 patients hospitalized with acute heart failure (AHF), we systematically evaluated the prognostic value of early biomarkers. The most robust and consistent predictor of in-hospital deterioration was lactate, which remained significant across all models, sensitivity analyses, and subgroups. A clear dose–response relationship was observed: nearly 86% of patients with lactate >4 mmol/L experienced deterioration, compared with 40% among those with lactate ≤2 mmol/L. NT-proBNP showed predictive value only among men, while NLR was significant in non-HFrEF patients. Troponin I did not independently predict outcomes. These sex-stratified and HF-phenotype–specific associations did not show statistically significant sex × biomarker interactions and are therefore interpreted as exploratory signals rather than definitive evidence of biological effect modification. Overall model discrimination was modest (AUC ∼0.71–0.73), but calibration was excellent, indicating reliable probability estimates. Surprisingly, in our cohort, women experienced a slightly higher crude rate of in-hospital deterioration compared with men (48.4% vs. 45.7%, Supplementary Table S3). Although this difference did not reach statistical significance, several factors may explain the numerical imbalance. Women were older on average and more frequently had HFpEF, both of which are known to be associated with adverse in-hospital outcomes. These patterns are consistent with prior reports highlighting sex-related differences in HF phenotype and prognosis.

### 4.2 Comparison with previous studies

Our findings extend prior literature by demonstrating that lactate, a non-cardiac biomarker reflecting tissue hypoperfusion and metabolic stress, provides early prognostic information in unselected AHF patients (15). In addition to cardiogenic shock, lactate can be elevated in sepsis, pneumonia, hypoxia, β-agonist use, and hepatic dysfunction, so in our composite endpoint—dominated by mechanical ventilation—it likely reflects global acute illness severity rather than heart-failure–specific pathophysiology alone. Previous studies in cardiogenic shock and critical illness have linked hyperlactatemia with poor prognosis; our results confirm its value in a broader AHF context. NT-proBNP, while widely established for HF diagnosis and long-term prognosis, did not independently predict short-term deterioration after adjustment, except in men. This sex difference is consistent with reports suggesting sex-related variation in natriuretic peptide biology, influenced by myocardial remodeling and renal clearance. NLR, an inflammatory marker, was predictive only in non-HFrEF, aligning with evidence that systemic inflammation plays a larger role in HFmrEF/HFpEF than in HFrEF. Troponin I, although central in diagnosing myocardial injury, was not predictive of early deterioration, suggesting its utility may be greater for long-term outcomes rather than acute risk. In this AMI-enriched cohort, troponin I concentrations were substantially higher in men than in women, whereas lactate medians were similar between sexes (Table 1). A plausible explanation is that troponin primarily reflects the extent of focal myocardial necrosis, which may be greater in men because of a higher burden of obstructive coronary disease and larger infarct size, while lactate integrates global hemodynamic compromise and systemic physiological stress at the time of sampling. Early reperfusion and standardized acute management may therefore attenuate potential sex differences in lactate despite marked divergence in troponin levels. Prior work has also linked inflammatory biomarkers to hemodynamic deterioration and shock, with some studies suggesting that women may be more prone to adverse circulatory trajectories; our sex-specific trends are directionally compatible with these data but clearly underpowered and should be regarded as hypothesis-generating rather than confirmatory (16, 17). Beyond biological sex, gender-related factors such as differences in smoking patterns, health-care–seeking behavior, treatment delay, and adherence to medical therapy may also contribute to the observed male–female differences in biomarker profiles and outcomes. These social and behavioral dimensions were not systematically captured in our dataset (beyond basic smoking and alcohol status), so we could not formally disentangle sex from gender effects, which should be examined in future prospective studies.

### 4.3 Clinical implications

These results highlight lactate as a readily available, inexpensive biomarker with strong clinical utility for early risk stratification in AHF. Measuring lactate at admission may help identify high-risk patients requiring close monitoring or escalation to advanced therapies, such as mechanical ventilation or ECMO. However, because mechanical ventilation comprised the majority of deterioration events and lactate can be driven by non-cardiac factors, its prognostic value in our study likely reflects overall acute illness severity and cardiopulmonary compromise rather than heart failure–specific risk alone. The exploratory sex- and phenotype-specific findings for NT-proBNP and NLR suggest that precision medicine approaches in HF may be valuable, but larger, adequately powered cohorts are needed to confirm true effect modification. Together, these findings suggest that integrating lactate with traditional biomarkers could enhance current triage and management strategies in AHF. Our findings could also inform the development of parsimonious risk scores that combine lactate with a small number of routinely collected variables to support early decision-making in AMI-related AHF.

### 4.4 Strengths and limitations

This study has several strengths. First, it leveraged systematic EMR-based patient identification across 15 years, ensuring a large and clinically relevant cohort. Second, strict inclusion criteria enhanced diagnostic specificity, and detailed manual verification minimized data errors. Third, the analysis incorporated a broad biomarker panel—NT-proBNP, troponin, lactate, and NLR—capturing complementary cardiac, metabolic, and inflammatory pathways. Fourth, sex-stratified analyses were performed, which remain rare in AHF biomarker research and add novelty by highlighting potential sex differences. Fifth, the statistical approach was rigorous, employing penalized regression, bootstrap internal validation, and sensitivity analyses (winsorization, alternative endpoints), which reduce bias and improve robustness compared with conventional single-center retrospective studies. Finally, transparency in endpoint definition and event composition, including both strict and broad criteria as well as the breakdown of deterioration events, represents a methodological strength that enhances interpretability.

Nevertheless, important limitations should be acknowledged. First, this was a single-center study with a relatively small sample size, limiting external generalizability and statistical power in subgroups. Second, the majority of patients had AMI-related AHF, which may limit applicability to decompensated HF without AMI. Third, AHF diagnosis was based on retrospective EMR coding and clinician notes rather than standardized BNP thresholds or echocardiographic criteria. While we excluded cases with clear non-cardiac causes (e.g., COPD, pneumonia), some misclassification remains possible. Fourth, biomarkers were measured only once within the first 24 h, precluding assessment of dynamic changes. Fifth, outcomes were limited to in-hospital deterioration; longer-term prognostic implications remain unknown. Sixth, while lactate was consistently robust, some subgroup results (NT-proBNP in men, NLR in non-HFrEF) may reflect limited power rather than true biological differences. Seventh, our models were constrained by modest event-per-variable ratios, especially in subgroup analyses. Although penalized regression and bootstrap resampling improved stability, wide confidence intervals and discrepancies between unpenalized and penalized estimates indicate residual imprecision. Subgroup findings (e.g., sex-stratified and HF phenotype-specific effects) should therefore be considered exploratory and hypothesis-generating rather than definitive. Eighth, our composite endpoint combined mortality with advanced interventions, which may reflect different underlying processes. Detailed, standardized information on vasopressor and inotrope use was not consistently available across the 15-year EMR period, so these therapies could not be incorporated into the endpoint definition and the overall burden of hemodynamic deterioration may therefore be underestimated. While this approach captures clinically relevant deterioration, it may also dilute interpretability. Notably, mechanical ventilation comprised the majority of events, suggesting that our findings primarily reflect predictors of respiratory failure and escalation of support rather than mortality alone. In addition, we did not systematically capture detailed treatment patterns, such as guideline-directed medical therapy dosing, vasopressor/inotrope use, or ventilator strategies, so we could not assess how therapeutic variation might have modified biomarker–outcome relationships. Finally, residual confounding cannot be excluded, and prospective multicenter external validation in more diverse AHF populations—including non-AMI presentations—is required before these findings can be generalized or translated into routine clinical decision-making (18).

## 5 Conclusion

In this retrospective cohort of patients hospitalized with acute heart failure, we developed and tested an early biomarker model for predicting in-hospital deterioration. Among the four evaluated biomarkers, lactate emerged as the most consistent and independent predictor across all models, sensitivity analyses, and clinical subgroups, with a clear dose–response relationship to adverse outcomes in this single-center, AMI-enriched AHF cohort. Exploratory sex-stratified analyses revealed numerical, but not statistically significant, differences: in men, both NT-proBNP and lactate were associated with deterioration, whereas in women, no biomarker achieved statistical significance, although lactate showed a directional trend. Furthermore, NLR contributed prognostic value specifically in the non-HFrEF subgroup in exploratory models, reinforcing the potential relevance of phenotype-based interpretation while underscoring the limited power of subgroup analyses.

Together, these findings demonstrate that early biomarker assessment—particularly lactate—can enhance prediction of in-hospital deterioration in acute heart failure, while sex-specific and phenotype-specific patterns should be regarded as hypothesis-generating rather than definitive evidence of biological effect modification. Future prospective multicenter studies with larger cohorts are warranted to validate these observations and to refine sex- and phenotype-based biomarker strategies for clinical decision-making.

## Data Availability

The raw data supporting the conclusions of this article will be made available by the authors, without undue reservation.
