# Supplementary material for: Sex-stratified early biomarker model identifies lactate as the key predictor of in-hospital deterioration in acute heart failure
Source: Front Cardiovasc Med. 2026 Jan 20;13:1717901. doi: 10.3389/fcvm.2026.1717901 (PMC12864415; doi:10.3389/fcvm.2026.1717901)
Supplement: Supplementary file 1 [file Datasheet1.pdf]

Supplementary Material

1 Supplementary Tables

Table S1. Sex-stratified regression results for Model A (males).

Term	Coef	OR	p
**const**	-6.382726	0.0017	0.00345
**lg_ntprobnp**	1.055254	2.873	0.02896
**lg_tni**	0.302540	1.353	0.1986
**lg_nlr**	0.138239	1.148	0.8646
**lg_lact**	3.366643	28.981	0.00265

Table S1. In men, both NT-proBNP and lactate were significant independent predictors.

Table S2. Sex-stratified regression results for Model A (females).

Term	Coef	OR	p
**const**	-1.047628	0.351	0.4713
**lg_ntprobnp**	0.026354	1.027	0.9483
**lg_tni**	0.022801	1.023	0.8522
**lg_nlr**	0.626829	1.872	0.3185
**lg_lact**	1.414301	4.114	0.0909

Table S2. In women, no biomarker reached significance, though lactate trended.

Table S3. Model A ROC by sex.

Stratum	n	events	AUC_point	95% bootstrap CI
**Male**	81	37	0.788	[0.681, 0.884]
**Female**	62	30	0.647	[0.523, 0.765]
**Overall**	143	67	0.714	[0.629, 0.784]

Table S3. ROC curves of Model A stratified by sex. Model discrimination was higher in males (AUC 0.788) than in females (AUC 0.647), indicating possible sex-based differences in biomarker performance.

Table S4. Interaction tests for sex × biomarker terms.

Biomarker	interaction_coef	p_interaction	n
lg_ntprobnp	0.922105	0.1189	143
lg_tni	0.268241	0.2808	143
lg_nlr	0.163660	0.8590	143
lg_lact	1.630250	0.2276	143

Table S4. Interaction tests for sex × biomarker terms in predicting in-hospital deterioration. None reached statistical significance, supporting sex-stratified reporting for descriptive purposes only.

Table S5. Penalized regression and bootstrap analyses (overall).

Predictor	OR_point	95% CI low	95% CI high	p_boot	p_z_approx
lg_ntprobnp	1.529	0.907	2.923	0.120	0.147
lg_tni	1.071	0.876	1.334	0.450	0.509
lg_nlr	1.507	0.733	3.215	0.278	0.294
lg_lact	4.704	2.226	11.164	0.000	0.00016

Table S5. Across all models (overall + subgroups), lactate consistently emerges as the most reliable biomarker.

Table S6. Penalized regression and bootstrap analyses (HFrEF subgroup).

Predictor	OR_point	95% CI	p_boot
lg_ntprobnp	1.908	0.84 – 4.38	0.130
lg_tni	1.271	0.95 – 1.90	0.094
lg_nlr	0.626	0.25 – 1.54	0.314
lg_lact	4.239	1.72 – 8.65	0.000

Table S6. In HFrEF subgroup (n=54), lactate is the strong predictor. Others not significant.

Table S7. Penalized regression and bootstrap analyses (non-HFrEF subgroup).

Predictor	OR_point	95% CI low	95% CI high	p_boot
**lg_ntprobnp**	1.378	0.785	2.806	0.256
**lg_tni**	0.976	0.744	1.292	0.866
**lg_nlr**	2.392	1.088	6.430	0.034
**lg_lact**	2.799	1.442	6.548	0.004

Table S7. NLR may carry added predictive value in non-HFrEF patients, but not in HFrEF. lactate is also the strong predictor.

Table S8. Events-per-variable (EPV) calculations for overall and subgroup models.

Model / Stratum	n	events	params (incl. intercept)	EPV
**Overall (Model A)**	143	67	5	13.40
**Overall (Model B)**	143	67	8	8.38
**HFrEF (<40%) (Model A)**	54	24	5	4.80
**non-HFrEF (Model A)**	89	43	5	8.60
**AMI subset (Model A)**	—	—	5	0.00 (na)

Table S8. Events-per-variable (EPV) calculations for overall and subgroup models. Low EPV values (<10) indicate limited stability, especially in subgroup analyses.

Table S9. Missingness of predictors and outcomes (supplementary).

Predictor	Variable name shown in tables	% missing
**NT-proBNP (log10)**	lg_ntprobnp	0.0
**Troponin I (log10)**	lg_tni	0.0
**Neutrophil:Lymphocyte ratio (log10)**	lg_nlr	0.0
**Lactate (log10)**	lg_lact	0.0
**Age**	age	0.0
**Creatinine**	creatinine / lg_creat	0.0
**HF phenotype (binary)**	hf_type_bin	0.0

Table S9. Missingness (%) for predictors in Models A and B. No missing data were observed, supporting a complete-case analysis (n=143).

Table S10. Winsorized Model A (99th percentile).

Term	Coefficient	Adjusted OR	p-value
**const**	-2.933349	0.0532	0.0120
**lg_ntprobnp**	0.460236	1.5844	0.1300
**lg_tni**	0.070586	1.0731	0.4795
**lg_nlr**	0.490571	1.6332	0.3064
**lg_lact**	2.174498	8.7978	0.0011

Table S10. Multivariable logistic regression (Model A) results after winsorizing biomarker values at the 99th percentile. Findings confirm the robustness of lactate as the only independent predictor of in-hospital deterioration.

Table S11. Original vs winsorized Model A comparison.

Term	Original Model A — Coef	Original Model A — OR (95% CI)	Original Model A — p	Winsorized Model A (99th pct cap) — Coef	Winsorized Model A — OR	Winsorized Model A — p
**const**	-2.932498	0.053264 (0.005410 – 0.524441)	0.011968	-2.933349	0.053219	0.012029
**lg_ntprobnp**	0.460968	1.585608 (0.874516 – 2.874909)	0.128934	0.460236	1.584449	0.129990
**lg_tni**	0.070897	1.073471 (0.882939 – 1.305119)	0.476991	0.070586	1.073137	0.479535
**lg_nlr**	0.484239	1.622939 (0.637265 – 4.133178)	0.309973	0.490571	1.633249	0.306360
**lg_lact**	2.167667	8.737873 (2.373903 – 32.162395)	0.001113	2.174498	8.797767	0.001073

Table S11. Comparison of original vs. winsorized Model A (99th percentile cap). Results were consistent across approaches, confirming robustness of lactate as the only significant biomarker.

Table S12. Winsorized Model A with bootstrap resampling.

Term	Coefficient (point)	OR (point)	95% bootstrap CI for OR	p_boot (two-sided)	p_z_approx	bootstrap SD (coef)
**const**	-2.933349	0.053	0.003 – 0.502	0.005	0.0262	1.319238
**lg_ntprobnp_win**	0.460236	1.584	0.868 – 3.335	0.133	0.1786	0.342131
**lg_tni_win**	0.070586	1.073	0.881 – 1.335	0.438	0.5048	0.105827
**lg_nlr_win**	0.490571	1.633	0.636 – 5.192	0.296	0.3508	0.525789
**lg_lact_win**	2.174498	8.798	2.960 – 57.399	0.000	0.0050	0.774430

Table S12. Winsorized Model A (99th percentile cap) with bootstrap inference (2,000 resamples). Lactate remained the only significant biomarker, confirming stability of findings.

2 Supplementary Figures

Figure S1. Histograms of log10-transformed biomarkers (NT-proBNP, troponin I, NLR, lactate).

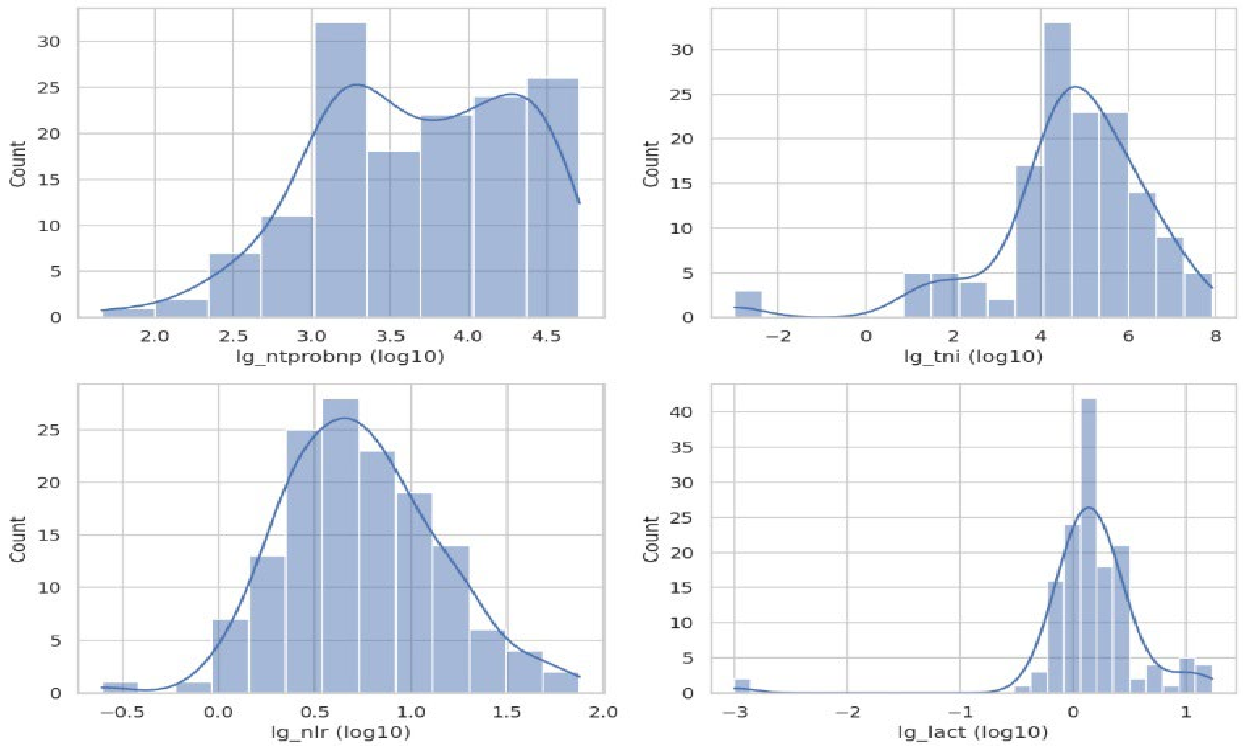

Figure S1. Histograms of log10-transformed biomarkers: NT-proBNP, troponin I, neutrophil-to-lymphocyte ratio (NLR), and lactate. Despite transformation, troponin and lactate remain right-skewed with potential outliers, supporting the use of sensitivity analyses.

Figure S2. Sex-stratified ROC curves for Model A.

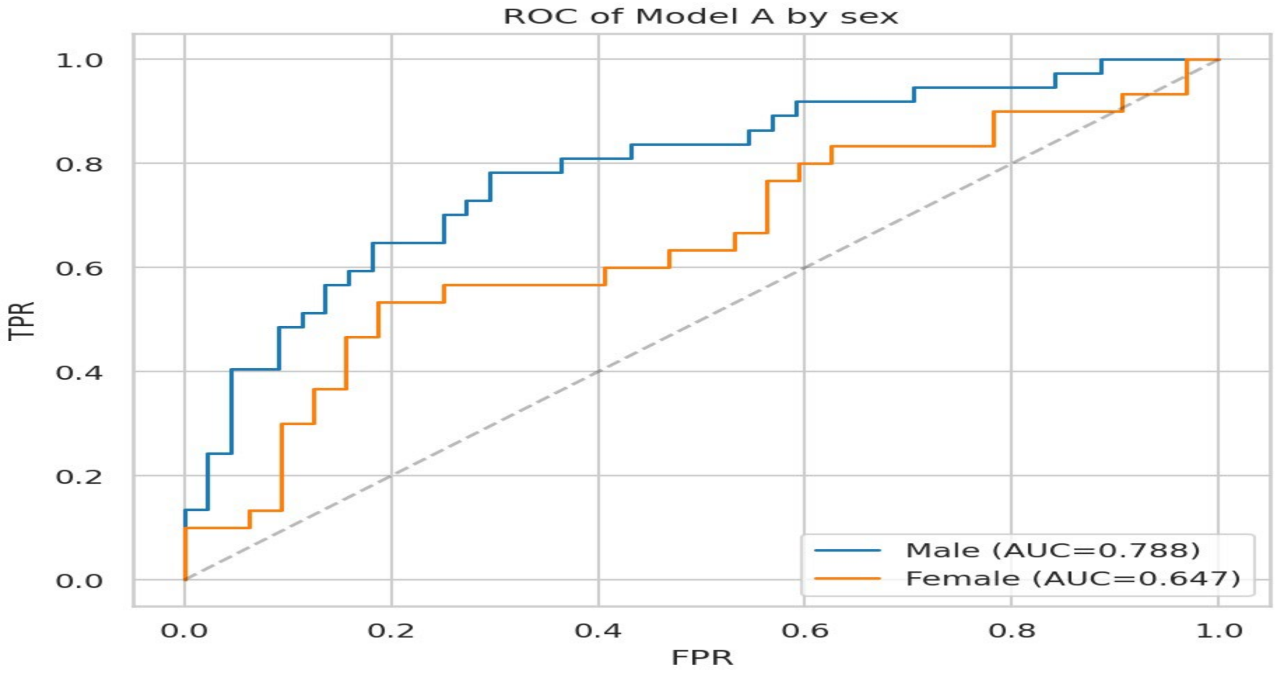

Figure S2. Model discrimination is higher in males than females. Interaction tests were non-significant statistically, but the stratified results are informative.

Figure S3. Penalized logistic regression with bootstrap (overall and subgroups).

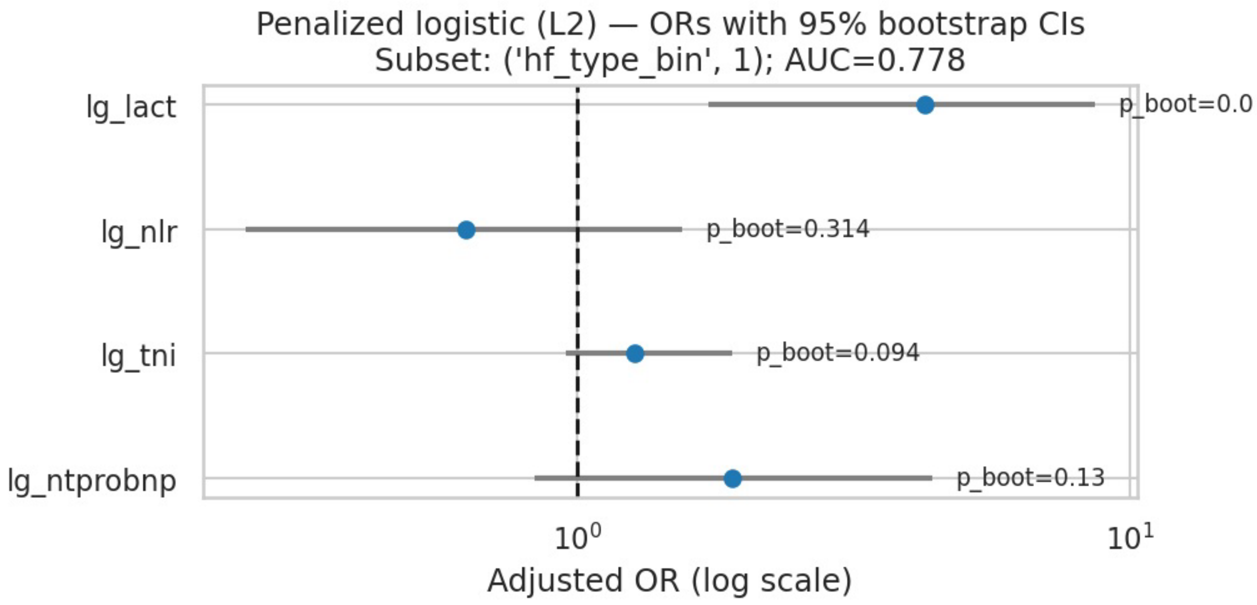

Figure S3. Lactate remained the only robust and consistent predictor of in-hospital deterioration. NLR showed additional predictive value in the non-HFrEF subgroup.

Figure S4. Calibration plot for AMI ordinal model

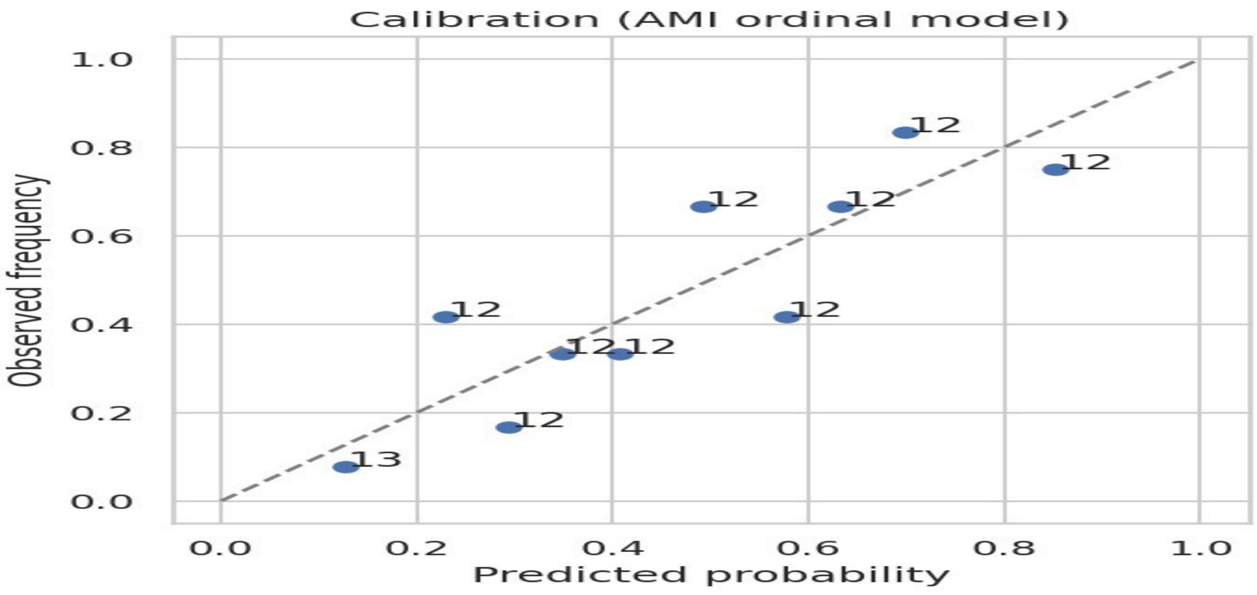

Figure S4. Calibration plot for AMI ordinal model (predicted vs observed by bins).

Figure S5. Adjusted ORs and ROC analyses in the AMI subset with Killip classification.

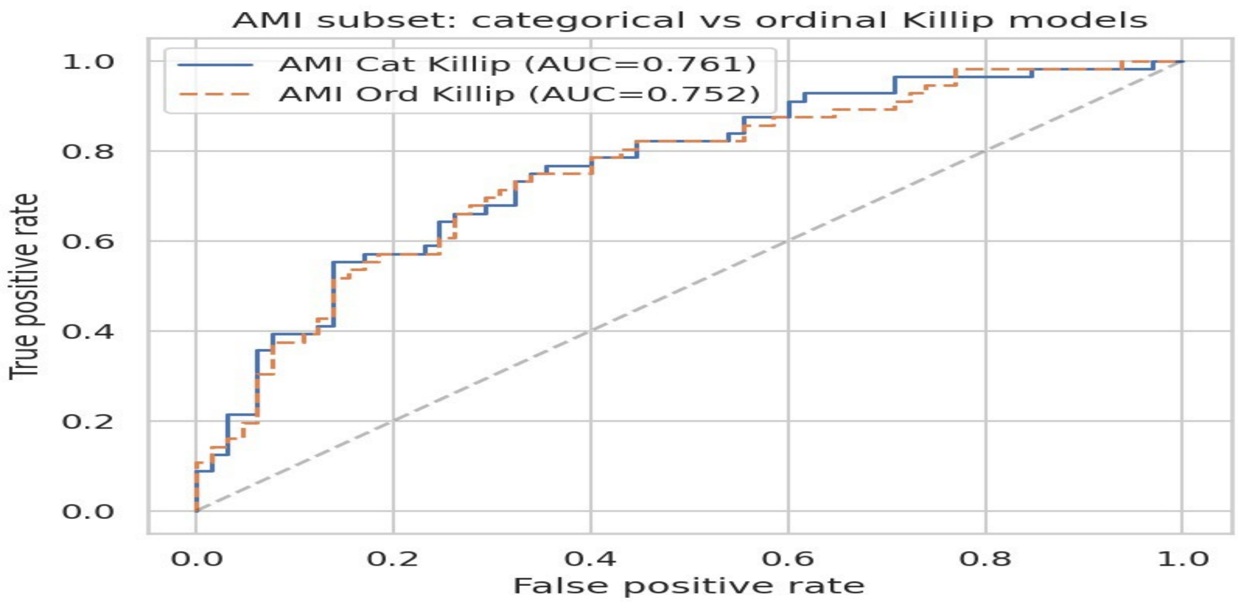

Figure S5. Lactate remained the most predictive biomarker after adjusting for Killip class. Model discrimination was good (AUC 0.75–0.76).
